# Advances in DNA metabarcoding for food and wildlife forensic species identification

**DOI:** 10.1007/s00216-016-9595-8

**Published:** 2016-05-13

**Authors:** Martijn Staats, Alfred J. Arulandhu, Barbara Gravendeel, Arne Holst-Jensen, Ingrid Scholtens, Tamara Peelen, Theo W. Prins, Esther Kok

**Affiliations:** RIKILT Wageningen UR, P.O. Box 230, 6700 AE Wageningen, The Netherlands; Naturalis Biodiversity Center, Sylviusweg 72, P.O. Box 9517, Leiden, The Netherlands; Norwegian Veterinary Institute, Ullevaalsveien 68, P.O. Box 750, Sentrum, 0106 Oslo Norway; Dutch Customs Laboratory, Kingsfordweg 1, 1043 GN Amsterdam, The Netherlands

**Keywords:** Endangered species, Next-generation sequencing, Wildlife forensic samples, Cytochrome *c* oxidase I, Convention on International Trade of Endangered Species

## Abstract

Species identification using DNA barcodes has been widely adopted by forensic scientists as an effective molecular tool for tracking adulterations in food and for analysing samples from alleged wildlife crime incidents. DNA barcoding is an approach that involves sequencing of short DNA sequences from standardized regions and comparison to a reference database as a molecular diagnostic tool in species identification. In recent years, remarkable progress has been made towards developing DNA metabarcoding strategies, which involves next-generation sequencing of DNA barcodes for the simultaneous detection of multiple species in complex samples. Metabarcoding strategies can be used in processed materials containing highly degraded DNA e.g. for the identification of endangered and hazardous species in traditional medicine. This review aims to provide insight into advances of plant and animal DNA barcoding and highlights current practices and recent developments for DNA metabarcoding of food and wildlife forensic samples from a practical point of view. Special emphasis is placed on new developments for identifying species listed in the Convention on International Trade of Endangered Species (CITES) appendices for which reliable methods for species identification may signal and/or prevent illegal trade. Current technological developments and challenges of DNA metabarcoding for forensic scientists will be assessed in the light of stakeholders’ needs.

## Introduction

Genetic identification of species plays a key role in the investigation of illegal trade of protected or endangered wildlife [1] and in the detection of species mislabelling and fraud in the food industry [2]. Currently, DNA barcoding is an established molecular technique that is used for differentiating and assigning taxonomy to species using standardized short DNA sequences (Box 1). Application of DNA barcoding for food authentication has gained much attention because of food safety concerns, including incorrect food labelling, food substitutions or food contamination [3–5]. DNA barcoding has been effective in the traceability of many processed food products in particular seafood and meat products [2]. For instance, DNA barcoding has made an impact by demonstrating widespread mislabelling or substitution of fish and seafood products in markets and restaurants in New York (USA) and Canada [4, 5]. Proper identification of species present in food and food supplements is of vital importance to protect consumers against potential food adulteration, ingredient mislabelling or food poisoning. Given its utility, DNA barcoding is being used by the US Food and Drug Administration as a replacement for the time-consuming technique of protein isoelectric focusing for fish and fish products [6].

Another established application of DNA barcoding to forensic science is in investigations of wildlife crimes such as illegal collection and trade of flora and fauna. More than 35,000 species of flora and fauna are categorized as endangered by the Convention on International Trade in Endangered Species of Wild Fauna and Flora (CITES). Protected species are listed in Appendices I, II and III, according to how severe a certain population is threatened by extinction [7]. Besides the regulated legal trade, a significant portion of the trade in endangered flora and fauna is illegal. In the European Union (EU), enforcement is mainly focused at the borders, where illegally imported wildlife products, plants or animals will be seized by customs and CITES authorities. The mailings on the EU-TWIX (European Union Trade in Wildlife Information eXchange; www.eutwix.org) network of wildlife-protecting enforcement bodies are very clear; seizures of wildlife and products containing wildlife are everyday practice. In some cases, the species identification of seized specimens is not very difficult, because the specific morphological characteristics can be readily observed, though often requiring taxonomic expertise for decisive identification. Identification will be more difficult when only parts of an animal or plant without distinctive morphological characteristics are present, or when plant or animal parts have been pulverized and have become ingredients of food supplements (e.g. traditional medicines, TMs). Currently, CITES lists species encompassing a wide diversity of species of terrestrial plants such as cycads, cacti and orchids, in addition to vertebrates such as fish, amphibians, reptiles, birds and mammals, and invertebrates such as lobsters, crabs and corals [8]. Customs laboratories will obviously benefit from applying standardized, fast and reliable methods when dealing with samples of which no a priori knowledge on the species composition is known. It is these benefits that have made DNA barcoding the method of choice for customs laboratories when trying to establish the presence of biological material from endangered species within processed products [9–12].

A complicating aspect for DNA barcoding in the analysis of food supplements such as TMs and other mixed products is that they are composed of more than one ingredient. Such samples often contain multiple species that can only be efficiently analysed if multiple DNA barcode templates can be sequenced in parallel, something that next-generation sequencing (NGS) technologies do effectively [13]. Current NGS platforms yield millions of DNA reads in a relatively short period of time, and the sequencers’ performance improves every year [14]. NGS combined with DNA barcoding is referred to as metabarcoding [15]. Metabarcoding thus uses universal PCR primers to mass-amplify one or more taxonomically informative targets. The prefix ‘meta’ refers to the collection of barcode sequences from different species. The general strategy consists of (1) extracting DNA from food or (wildlife) forensic samples, (2) amplifying a specific DNA barcode or other target region of taxonomic value, (3) sequencing the corresponding DNA amplicons using NGS technology, (4) analysing the sequences using appropriate bioinformatics pipelines, (5) identifying the species in the sample from which DNA has been extracted and (6) screening for CITES species among these [16, 17]. Metabarcoding has been applied in many diverse environmental samples, such as faeces [15], soil [18], marine water [19] and bulk samples of tropical arthropods [20]. However, there are only a few published applications of metabarcoding to food and (wildlife) forensic samples. Coghlan et al. [21] demonstrated the power of metabarcoding in detecting species in complex traditional Chinese medicine (TCM) samples presented in the form of powders, crystals, capsules, tablets and herbal tea. Their screening revealed that some of the TCM samples contained CITES listed species, including the Asiatic black bear (*Ursus thibetanus*) and the Saiga antelope (*Saiga tatarica*), as well as unlisted ingredients, and potentially toxic and allergenic plants. Cheng et al. [22] performed metabarcoding analyses on well-defined TCM preparations based on a six-herb formula named Liuwei Dihuang Wan, which is widely used in China. They concluded that there are significant differences in quality and safety among commercial TCM preparations, as the unlisted species *Senna obtusifolia* was identified in some preparations that may potentially pose safety risks to consumers. Tillmar et al. [23] developed a metabarcoding method for the identification of species of mammals in human forensic tissues, with which the presence of low quantities of DNA from the genus *Canis* could be identified.

Although metabarcoding may seem easy to apply, researchers often face limitations in obtaining a representative assessment of species composition. First, different preprocessing conditions and production procedures from samples with different composition and matrices (e.g. TMs and other processed and complex products) may result in highly variable DNA quality and concentration. DNA integrity has a significant influence on the effectiveness of the metabarcoding and other molecular methodologies for species identification [22, 24, 25]. Secondly, while there are many bioinformatics methods available for the analysis of metabarcoding data, the discriminating power of these methods is directly related to prior choices on barcode marker and reference database composition [26, 27]. PCR bias caused by variable primer–template mismatches across species may limit the quantitative potential of DNA metabarcoding and may cause species to be missed [28, 29]. Furthermore, DNA metabarcoding wholly relies on the presence of high-quality barcode sequence reference databases that are based on good taxonomy and barcode coverage. The goal of this paper is to review the advancements and current practices of plant and animal metabarcoding, with an emphasis on complex food and forensic wildlife samples for identifying, in particular, species listed by CITES. This effort is complementary to recent work focussing on metabarcoding for biodiversity assessments in environmental samples [17, 30–32] and an extension of the work on DNA barcoding of food and forensic samples [1–3, 33–36]. This overview will address the opportunities and challenges that must be faced to allow the customs laboratories and other routine laboratories to perform efficient and reliable metabarcoding analysis that can broadly identify any species present in a sample under investigation.

Box 1. DNA barcoding and the International Barcode of Life project (iBOL)

**Table:** 

DNA barcoding is a rapid method of differentiating and assigning taxonomy to species using standardized short DNA sequences. For animals, the most commonly used sequence is a 658-bp (base pair) region of the mitochondrial cytochrome *c* oxidase subunit I gene (COI, COX1, CO1). DNA barcoding allows for fast, reliable, automatable and cost-effective species identification by users with little or no taxonomic experience [37]. Identifications are usually made by comparing unknown sequences against known species DNA barcodes via alignment searching (BLAST) [38] or distance-based tree construction [39]. A suitable barcode for identification at the species level should be sufficiently variable between species (typically at least 3 % difference between closely related species but this may vary amongst taxonomic groups) and display either low or no intraspecific variations. Also, barcodes should be widely studied for a large number of species to enable comparison of the nucleotide sequence from an unknown sample with reference sequences in a database. Accurate species identification wholly relies on the taxonomic coverage of barcodes in a reference database. If the query sequence lacks a conspecific (belonging to the same species) target sequence in the database, species-level barcoding-based identification of the query will fail. Instead, the closest matches in the database may be identified and the sample barcode scored as a “new” taxon (operational taxonomic unit, OTU). From a practical point of view, therefore, DNA barcoding requires a comprehensive reference database. Such reference data sets are being assembled by the barcoding campaigns initiated by the International Barcode Of Life project (iBOL; www.ibol.org), resulting in considerably improved species coverage for target taxa of such DNA barcoding campaigns [40]. Official barcode sequences generated by the iBOL initiatives are deposited and organized in the Barcode Of Life Data (BOLD) Systems (http://boldsystems.org; [41]). BOLD is a large-scale and rigorously curated DNA barcode storage database, and most of the sequence information contained within BOLD has been derived from voucher specimens with authoritative taxonomic identifications. Barcoding campaigns focussing on fish, birds, mammals, insects and fungi have been initiated e.g. the Fish Barcode of Life Initiative (FISH-BOL, www.fishbol.org), the Marine Barcode Of Life Initiative (MarBOL, www.marinebarcoding.org), the Shark Barcode Of Life project (SharkBOL; www.sharkbol.org) and the Barcode of Wildlife Project (BWP; www.barcodeofwildlife.org). For plants there are initiatives to barcode e.g. the world’s tree species in TreeBOL and grasses and grass-like plants in GrassBOL. Barcodes and a variety of alternative taxonomically informative genes that have been generated from general scientific research are deposited in the International Nucleotide Sequence Database Collaboration (INSDC) and can be used for taxonomic assignment in barcoding studies. The iBOL initiative aims to create a database of 5 million standardized DNA sequences, which can be used to identify 500,000 species, by 2015. Scientific literature on the utility of DNA barcoding in the recognition, discrimination and discovery of plant and animal species has been reviewed extensively by Savolainen et al. [42], Kress and Erickson [43], Bucklin et al. [44], Hollingsworth et al. [26], Fazekas et al. [45], Ortea et al. [33], Nicolè et al. [34], Bhargava and Sharma [46], Kvist [47] and Sandionigi et al. [48].	

### DNA extraction and DNA integrity

The initial sample preparation and extraction step in the analysis of DNA from food products is probably the most crucial step in the process of species identification in complex forensic samples. This step can be very difficult to standardize and optimize because of the complexity and diversity of the matrices encountered, each presenting different problems. For instance, it can be difficult to ensure that a representative sample is obtained from heterogeneous samples that are composed of many ingredients (e.g. TMs), and in such cases sufficient homogenization is particularly critical prior to DNA extraction. Forensic samples, such as food samples and TM may contain only very low amounts of DNA or contain ingredients that have been subjected to various treatments during the production process (e.g. cooking, high pressure, pH modification, grinding or drying), which may cause the DNA to be highly degraded [22, 49–51]. Furthermore, failure to eliminate potential inhibitory components and interfering substances from the material under investigation (e.g. protein, lipids, polyphenols, polysaccharides) may severely influence PCR analysis. Needless to say, any factor that may contribute to downstream bias needs to be minimized.

Different DNA extraction methods which can be used for analysis of forensic samples are now available; extraction is either based on in-house developed protocols or commercially available kits. Commercial kits offer a means for standardizing DNA extraction from forensic samples, as the protocol can be easily implemented in any laboratory. However, in many laboratories user-specific protocols have been developed to improve DNA extraction efficiency on a case-by-case basis. DNA extraction using cetyltrimethylammonium bromide (CTAB) extraction buffer combined with additional silica or a resin-based purification step are efficient for a wide range of plants and plant-derived products, in particular for separation of polysaccharides from DNA [25, 51, 52]. Ivanova et al. [53] developed a cost-efficient and automation-friendly DNA extraction protocol for animal tissues that consists of a tissue lysis step (SDS and proteinase K) followed by silica-based purification of DNA using inexpensive glass fibre filtration plates. The latter method has been used to process thousands of animal species at the Canadian Centre for DNA Barcoding (CCDB) as part of the iBOL initiative. Despite these efforts in standardizing the DNA extraction method, the most suitable method is generally strongly dependent on the matrix, and there is no “universal” method that could be used for all food and (wildlife) forensic samples [49].

As suspect samples may often contain degraded DNA, it is a requirement that metabarcoding methods are able to identify species on the basis of short DNA sequences that may still be present in highly processed materials [51]. In such forensic samples, DNA degradation often prevents the amplification of PCR fragments longer than approximately 300 bp [24, 25, 54, 55]. The use of shorter barcode regions, so-called mini-barcodes, may overcome this problem. Owing to their reduced size, mini-barcodes are often amplified with higher efficiency in degraded samples than standard, full-length barcodes, which are typically 650–900 bp in length [25, 56]. On the other hand, the rate of taxonomic discrimination is generally positively correlated with the length of the mini-barcode. The use of universal mini-barcodes that will only allow identification of taxa above the species level, as a result of saturation of the taxonomic discrimination, should generally be avoided unless identification at the genus or family level is warranted.

### Animal DNA barcodes and mini-barcodes

For animals, the standard barcode is a 658-bp region in the gene encoding mitochondrial cytochrome *c* oxidase I (COI or COX1, CO1) [57]. COI has long been used in animal molecular systematics to study relationships of closely related species because of its high level of interspecific variation [58]. Its popularity within the barcoding community is clearly reflected in the large public databases such as National Centre for Biotechnology Information (NCBI) GenBank (www.ncbi.nlm.nih.gov/genbank) and BOLD (Box 1). Universal primer sets for amplifying the COI barcode across major taxonomic groups have been developed by Ivanova et al. [59], and primer cocktails have been reported that are effective in fish, mammals, amphibians and reptiles (Table 1). A good discriminatory power in the identification of birds (98–100 % identification success rate [39]), fish (93–98 % identification success rate [64]), spiders (100 % identification success rate [78]), butterflies (97.9 % identification success rate [63]) and reptiles (72.7–100 % identification success rate [61]) has been shown for the COI barcode.

**Table 1:** Non-exhaustive list of primers for amplifying animal DNA barcodes and mini barcodes

DNA marker	Target taxonomic group	Primer name	Primer sequences (5'–3')	Amplicon length (bp)	Remark	Reference
COI	Various phyla	LCO1490	GGTCAACAAATCATAAAGATATTGG	648		Folmer et al. [60]
		HCO2198	TAAACTTCAGGGTGACCAAAAAATCA			
COI	Reptiles	RepCOI-F	TNTTMTCAACNAACCACAAAGA	664		Nagy et al. [61]
		RepCOI-R	ACTTCTGGRTGKCCAAARAATCA			
COI-1	Birds	BirdF1	TTCTCCAACCACAAAGACATTGGCAC	648	Forward	Hebert et al. [39]
		BirdR1	ACGTGGGAGATAATTCCAAATCCTG		Reverse	
COI	Insects and amphibians	LepF1	ATTCAACCAATCATAAAGATATTGG	648		Hebert et al. [62]
		LepR1	TAAACTTCTGGATGTCCAAAAAATCA			
		MLepF1	GCTTTCCCACGAATAAATAATA (use with LepR1)			Hajibabaei et al. [63]
		MLepR1	CCTGTTCCAGCTCCATTTTC (use with LepF1)			
COI-2	Mammals, fish, reptiles and amphibians	LepF1_t1	TGTAAAACGACGGCCAGTATTCAACCAATCATAAAGATATTGG	648	M13-tailed cocktail; mix ratio 1:1:1:3:1:1:1:3	Ivanova et al. [59]
		VF1_t1	TGTAAAACGACGGCCAGTTCTCAACCAACCACAAAGACATTGG			
		VF1d_t1	TGTAAAACGACGGCCAGTTCTCAACCAACCACAARGAYATYGG			
		VF1i_t1	TGTAAAACGACGGCCAGTTCTCAACCAACCAIAAIGAIATIGG			
		LepR1_t1	CAGGAAACAGCTATGACTAAACTTCTGGATGTCCAAAAAATCA			
		VR1d_t1	CAGGAAACAGCTATGACTAGACTTCTGGGTGGCCRAARAAYCA			
		VR1_t1	CAGGAAACAGCTATGACTAGACTTCTGGGTGGCCAAAGAATCA			
		VR1i_t1	CAGGAAACAGCTATGACTAGACTTCTGGGTGICCIAAIAAICA			
COI-3	Fish and mammals	VF2_t1	TGTAAAACGACGGCCAGTCAACCAACCACAAAGACATTGGCAC		M13-tailed cocktail; mix ratio 1:1:1:1	Ivanova et al. [59]
		FishF2_t1	TGTAAAACGACGGCCAGTCGACTAATCATAAAGATATCGGCAC			
		FishR2_t1	CAGGAAACAGCTATGACACTTCAGGGTGACCGAAGAATCAGAA			
		FR1d_t1	CAGGAAACAGCTATGACACCTCAGGGTGTCCGAARAAYCARAA			
COI	Sharks	FishR2	ACTTCAGGGTGACCGAAGAATCAGAA	550		Ward et al. [64]
		Shark-int	ATCTTTGGTGCATGAGCAGGAATAGT			
COI	Echinodermata phylum	COIceF	ACTGCCCACGCCCTAGTAATGATATTTTTTATGGTNATGCC	>550		Hoareau and Boissin [65]
		COIceR	TCGTGTGTCTACGTCCATTCCTACTGTRAACATRTG			
COI	Universal animal mini-barcode	mlCOIintF	GGWACWGGWTGAACWGTWTAYCCYCC	313		Leray et al. [66]
		jgHCO2198	TAIACYTCIGGRTGICCRAARAAYCA			Geller et al. [67]
COI	Universal animal mini-barcode	Uni-MinibarR1	GAAAATCATAATGAAGGCATGAGC	130		Meusnier et al. [24]
		Uni-MinibarF1	TCCACTAATCACAARGATATTGGTAC			
cyt*b*	Universal mammal	L14724	CGAAGCTTGATATGAAAAACCATCGTTG	1140	Full-length cyt*b*	Irwin et al. [68]
		H15915	AACTGCAGTCATCTCCGGTTTACAAGAC			
cyt*b*	Universal fish	FishcytB-F	ACCACCGTTGTTATTCAACTACAAGAAC	750	cyt*b*-5' fragment	Sevilla et al. [69]
		CytB1-5R	GGTCTTTGTAGGAGAAGTATGGGTGGAA			
cyt*b*	Universal vertebrate animal mini-barcode	L14816	CCATCCAACATCTCAGCATGATGAAA	357		Parson et al. [70]
		H15173	CCCCTCGAATGATATTTGTCCTCA			
cyt*b*	Universal animal mini-barcode	L14841	AAAAAGCTTCCATCCAACATCTCAGCATGATGAAA	307		Kocher et al. [71]
		H15149	AAACTGCAGCCCCTCAGAATGATATTTGTCCTCA			
16S	Universal animal	16sar-L	CGCCTGTTTATCAAAAACAT	500–650	Forward	Palumbi [72]
		16sbr-H	CCGGTCTGAACTCAGATCACGT		Reverse	
16S	Universal animal mini-barcode	16S-forward	AYAAGACGAGAAGACCC	250		Sarri et al. [73]
		16S-reverse	GATTGCGCTGTTATTCC			
16S	Fish, cephalopods and crustaceans	16S1F	GACGAKAAGACCCTA	250		Deagle et al. [74]
		16S2R	CGCTGTTATCCCTADRGTAACT			
16S	Universal mammal mini-barcode	16S-forward	GACGAGAAGACCCTATGGAGC	100		Tillmar et al. [23]
		16S-reverse	TCCGAGGTCACCCCAACCTCCG			
16S	Universal vertebrate mini-barcode	L2513	GCCTGTTTACCAAAAACATCAC	244		Kitano et al. [75]
		H2714	CTCCATAGGGTCTTCTCGTCTT			
16S	Universal animal mini-barcode	16S-HF	ATAACACGAGAAGACCCT	80–125		Horreo et al. [76]
		16S-HR1	CCCACGGTCGCCCCAAC			
		16S-HR2	CCCGCGGTCGCCCCAAC			
12S	Universal vertebrate mini-barcode	L1085	CCCAAACTGGGATTAGATACCC	215		Kitano et al. [75]
		H1259	GTTTGCTGAAGATGGCGGTA			
12S	Universal vertebrate mini-barcode	12SV5-F	TTAGATACCCCACTATGC	98		Riaz et al. [77]
		12SV5B2	TAGAACAGGCTCCTCTAG			

Despite its proven effectiveness, COI is not always suitable and effective for identifying all animal species. For endangered organisms such as sea snails (the mollusc class Gastropoda) and corals the COI barcoding region and other mitochondrial markers were found to offer insufficient resolution to allow for reliable discrimination between closely related species [79–82]. Using a DNA metabarcoding approach, Elbrecht et al. [29] demonstrated that species may go undetected in complex artificial mixtures of freshwater invertebrate taxa because of universal COI primer–template mismatches. The use of group-specific primers or alternative degenerate primers may prevent species from being missed using COI [28, 29].

The traceability of mammalian meat including meat of ranched and hunted game species heavily relies on the use of the mitochondrial cytochrome *b* (cyt*b*) region [2, 83]. The choice of cyt*b* instead of COI is due mainly to practical reasons. The early availability of universal primers for cyt*b* [63, 78], long before the use of COI became popular, led to the deposition of several thousand cyt*b* sequences of a large range of edible mammalian species in public databases. Thus, its use became well established. Nonetheless, DNA barcoding based on COI has also proven effective in the identification of edible meat, including bush meat species [2, 83–86]. The FishTrace consortium (www.fishtrace.org) has promoted the use of cyt*b* through the development of universal cyt*b* primers for teleost fish species and the release of validated sequence data of many hundreds of European marine fish species [69].

Additional activities have taken place in finding suitable short DNA regions and related PCR primers for barcoding of species in widely diverse food and forensic samples, but so far no true mini-barcode standard has been adopted. Efforts in designing short broad coverage COI barcodes (i.e. mini-barcodes) to accommodate identification of a diversity of animal species in samples with degraded DNA has proven to be difficult. The use of the 130-bp COI mini-barcode primers designed by Meusnier et al. [24] has been limited [87], because the priming sites in the COI gene used in the mini-barcode design are not sufficiently conserved to cover a broad range of taxa [27, 66]. Leray et al. [83] have adopted a thorough approach and used the COI barcodes provided by the Moorea BIOCODE project, an “All Taxa Biotic Inventory” (www.mooreabiocode.org), consisting of more than 64,000 sequences across all phyla to design conserved universal COI mini-barcoding primers to target a 313-bp region. The newly designed primers were reported to perform well across metazoan diversity, with a higher success rate than the versatile primer sets traditionally used for DNA barcoding, i.e. the “Folmer primers” HCO2198 and LCO1490 [60] (Table 1).

Mitochondrial cyt*b*, 12S and 16S rRNA genes are the most commonly used genetic markers for species discrimination in degraded samples [88]. Universal primers for the amplification of short regions of cyt*b* have been developed for various animal taxa [70, 71]. Their use has been demonstrated in different problematic forensic samples that may contain degraded DNA including hair shafts, bones, feathers and meat products [70, 83].

Mini-barcodes based on the 12S and 16S rDNA mitochondrial genes have recently been demonstrated by several studies to be suited for identifying a wide range of animal species in environmental samples [89] and processed food and wildlife forensic products including TMs [21, 23, 73, 76]. The 12S and 16S rDNA contain internal regions that are strongly conserved across taxa, suitable for designing universal primers, alternated with short hypervariable regions that are species-specific. Sarri et al. [73] developed an approximately 250-bp barcode marker (Table 1) which allowed for the successful amplification of the 16S region across different sample types (e.g. cheese, processed meats, frozen fish fillets) and the correct identification of a wide range of animals in food products, including fishes, birds, reptiles, crustaceans and European mammals. Kitano et al. [75] developed 12S and 16S mini-barcodes for the identification of a large number of vertebrates (mammals, birds, reptiles, amphibians and fish). Similarly, Karlsson and Holmlund [88] used short 12S and 16S regions to identify a total of 28 different mammals including domestic and game species.

### Plant DNA barcodes and mini-barcodes

In plants, the COI gene and other mitochondrial regions are a poor choice for species identification because the mitochondrial genome in plants has evolved too slowly to allow it to be used for DNA barcoding [90]. Research into a COI analogue in plants has focused on the plastid genome, but the selection of a standard plant barcode marker has been complicated by the trade-off that arises between the high requirements of universality and high variability among plants [43]. So far, no single barcode marker has been found that is expected to discriminate all of the more than 200,000 species of plants. The Consortium for the Barcode Of Life (CBOL) plant working group has opted for the use of a core set of two (*rbc*L and *mat*K) coding sequences from plastids as the “core” DNA barcode (Table 2) [26]. The *rbc*L barcode consists of a 599-bp region at the 5' end of the gene. It is easy to amplify, sequence and align in most land plants, but it has only modest discriminatory power. Newmaster et al. [102] analysed over 10,000 *rbc*L sequences from GenBank and found that *rbc*L could discriminate samples in approximately 85 % of pairwise comparisons of congeneric species. The *mat*K barcode region consists of a ca. 841-bp region at the centre of the gene, which is one of the most rapidly evolving regions of the plastid genome. The *mat*K is perhaps the closest plant analogue to the COI animal barcode [103]. Ogden et al. [104] developed a single-nucleotide polymorphism (SNP) genotyping approach based on *mat*K DNA barcodes to distinguish between traded timber products of Ramin (*Gonystylus*) species, which are all CITES protected. Unfortunately, *mat*K can be difficult to amplify, particularly in non-angiosperms, because of the lack of sufficiently universal primers [92, 94].

**Table 2:** Non-exhaustive list of primers for amplifying plant DNA barcodes and mini-barcodes

DNA marker	Target taxonomic group	Primer name	Primer sequences (5'–3')	Amplicon length (bp)	Remark	Reference
*rbc*L	Universal plant	rbcL a-F	ATGTCACCACAAACAGAGACTAAAGC	654		Levin et al. [91]
		rbcL a-R	GTAAAATCAAGTCCACCRCG			Kress and Erickson [92]
*mat*K	Angiosperms & Gymnosperms	matK-KIM1R	ACCCAGTCCATCTGGAAATCTTGGTTC	656–889	Forward	Fazekas et al. [45]
		matK-KIM3F	CGTACAGTACTTTTGTGTTTACGAG		Reverse	
*mat*K	Angiosperms & Gymnosperms	matK-390f	CGATCTATTCATTCAATATTTC	656–889	Forward	Cuénoud et al. [93]
		matK-1326r	TCTAGCACACGAAAGTCGAAGT		Reverse	
*mat*K	Gymnosperms	NY552F	CTGGATYCAAGATGCTCCTT	656–889	Forward	Fazekas et al. [45]
		NY1150R	GGTCTTTGAGAAGAACGGAGA		Reverse	
*mat*K	Gymnosperms	matKpkF4	CCCTATTCTATTCAYCCNGA	656–889	Forward	Fazekas et al. [94]
		matKpkR1	CGTATCGTGCTTTTRTGYTT		Reverse	
nrITS2	Universal plant	S2F	ATGCGATACTTGGTGTGAAT		Forward	Chen et al. [95]
		ITS4	TCCTCCGCTTATTGATATGC		Reverse	White et al. [96]
nrITS2	Universal plant	S2F	ATGCGATACTTGGTGTGAAT	160–320	Forward	Chen et al. [95]
		S3R	GACGCTTCTCCAGACTACAAT		Reverse	
nrITS	Universal angiosperm	17SE	ACGAATTCATGGTCCGGTGAAGTGTTCG	800	Forward	Sun et al. [97]
		26SE	TAGAATTCCCCGGTTCGCTCGCCGTTAC		Reverse	
*trn*H-*psb*A	Universal plant	psbAF	GTTATGCATGAACGTAATGCTC	264–792		Sang et al. [98]
		trnH2	CGCGCATGGTGGATTCACAATCC			Tate and Simpson [99]
*trn*L (UAA)	Universal plant mini-barcode	g	GGGCAATCCTGAGCCAA	10–143	p-loop region of *trn*L	Taberlet et al. [100]
		h	CCATTGAGTCTCTGCACCTATC			
*trn*L (UAA)	Universal plant	c	CGAAATCGGTAGACGCTACG	767		Taberlet et al. [101]
		d	GGGGATAGAGGGACTTGAAC			
*trn*L (UAA)	Universal plant mini-barcode	c	CGAAATCGGTAGACGCTACG	250		Taberlet et al. [101]
		h	CCATTGAGTCTCTGCACCTATC			Taberlet et al. [100]

The two most widely used supplementary loci are the nuclear ribosomal ITS (nrITS) [95] and plastid intergenic spacer *psb*A-*trn*H region [105]. The nrITS region had previously been discounted as a standard DNA barcode because of concerns over paralogy and the presence of putative pseudogenes which led to sequencing difficulties in many plant groups [106]. However, the increased resolution of nrITS over plastid DNA barcodes in many studies suggested that it should continue to be explored as part of the plant DNA barcode [95, 107]. Some authors have noted that just using a subset of the ribosomal cassette (nrITS2) can lead to greater amplification and sequencing success compared to the entire nrITS region [95]. By testing the discriminating ability of nrITS2 in more than 6600 medicinal plants and closely related samples, Chen et al. [95] found that the rate of successful identification was 92.7 % at the species level, and they proposed that the nrITS2 region should be the standard barcode for investigating forensic samples containing medicinal plants. Newmaster et al. [102] used *rbc*L and nrITS2 DNA barcodes to highlight species substitution and contamination in herbal products.

The *psb*A-*trn*H region is straightforward to amplify across land plants and is one of the more variable intergenic spacers in plants [108]. It has been used successfully in a range of barcoding studies [109, 110]. One of the main concerns associated with the use of *psb*A-*trn*H as a standard barcode is the premature termination of sequence reads by mononucleotide repeats leading to unidirectional reads in up to 30 % of sequences [111].

In plants, the design of suitable universal mini-barcode markers has proven difficult. The length constraints to allow working with highly degraded DNA severely limit the taxonomic resolution of mini-barcodes compared to that of the 500- to 800-bp-long standardized barcodes (*rbc*L, *mat*K). Primers for the amplification of an approximately 180-bp region of chloroplast *rbc*L have been used, but in most cases this system only allows the identification of families, not genera or species [112]. Little [56] evaluated a variety of *rbc*L primers in silico and found the discriminatory power of the best *rbc*L mini-barcode to be less than 38.2 %. Taberlet et al. [100] used the chloroplast tRNA^Leu^ (UAA) intron sequences [*trn*L (UAA), 254–767 bp] and a shorter fragment of this intron (the P6-loop, 10–143 bp) for identifying plant species in processed food and ancient permafrost samples. The number of *trn*L (UAA) intron sequences available in databases is high, by far the most numerous among non-coding chloroplast DNA sequences. The *trn*L (UAA) region had overall low resolution. However, Taberlet et al. [100] concluded that only closely related species are not resolved and that the region can effectively be used to identify commonly eaten plants (e.g. potato, tomato, maize, but not almond). The *trn*L (UAA) has been extensively used in the food industry [113], forensic sciences [12] and diet studies based on faeces [114].

For some applications, a plant mini-barcode with relatively modest discriminatory power at the genus or higher taxonomic level can be useful. For example, it is often an entire genus or family that is listed by CITES, rather than individual plant species. For many plant families listed by CITES (e.g. Cycadaceae, Orchidaceae, Cactaceae, Euphorbia) identification to a larger group is therefore all that is required. This does not apply to all illegally traded plant genera though, such as tree ferns of the genus *Cibotium*, of which only *C. regale* is legally protected. In such cases, an alternative approach could be to design species-specific mini-barcodes to distinguish between closely related species from the CITES listed species, as was done for instance for *Rauvolfia serpentina* [12].

Box 2. Approximate number of sequences of DNA barcodes and other taxonomically informative genes available in GenBank (December 2014). GenBank sequences were retrieved with a query of the sequence annotations using the nucleotide database e.g. ‘COI’ OR ‘cytochrome *c* oxidase’ AND eukaryote‘. The query headers were then downloaded and additionally filtered using the GNU/Linux command line tools (e.g. awk and grep). The number of unique genera and species were estimated from the sequence annotations and should be considered only as an approximation.

**Table:** 

Number of barcoding sequences deposited in GenBank								
	COI	16S	cyt*b*	*mat*K	*rbc*L	*trn*L	*psb*A-*trn*H	nrITS
Approx. number of accessions	940,687	264,931	324,769	94,246	134,784	172,493	44,581	378,711
Approx. number of species	102,919	60,928	34,230	43,039	47,675	63,172	20,891	84,670
Approx. number of genera	30,923	21,691	10,822	8759	10,978	10,895	3836	14,338

### Sequencing of DNA barcodes using NGS technology

There are many excellent reviews on NGS platforms, and also their fundamentals and broad characteristics are described elsewhere [13, 14, 115, 116]. We will focus on the important steps in the NGS workflow and only provide a brief overview of NGS technologies relevant for DNA metabarcoding.

Early DNA metabarcoding studies have employed the 454 pyrosequencing technology of Roche because it was the first commercially available NGS system and because of its longer sequence read-outs allowing for a more informative fraction of DNA barcodes to be sequenced. Pyrosequencing has been used for DNA metabarcoding of raw materials of the diet of several animals [66, 114], environmental monitoring [15, 117, 118] and for analysing ancient DNA

Box 3. Benchtop next-generation sequencing system and their characteristics. Benchtop instruments are scaled-down, economical NGS platforms driven by the need for cheaper and faster sequencing, and which are suited for metabarcoding of typical food and forensic samples. The system specificities are listed with expected maximum performance by the beginning of 2016.

**Table:** 

Instrument	Company	Machine run time (h)	Reads/run	Read length (base)	Output
454 GS Junior Plus^a^	Roche	18	70,000	ca. 700	70 Mb
MiniSeq^b^	Illumina®	24	44–50 million	2 × 150	6.6–7.5 Gb
MiSeq^c^	Illumina®	56	44–50 million	2 × 300	13.2–15 Gb
NextSeq 500^d^	Illumina®	29	Up to 800 million	2 × 150	100–120 Gb
Ion PGM™ System^e^	Ion Torrent™	7.3	4–5.5 million	400	1.2–2.0 Gb
Ion Proton™ System^f^	Ion Torrent™	4	60–80 million	200	Up to 10 Gb

^a^Adopted from http://454.com/products/gs-junior-plus-system/index.asp. Roche announced that 454 sequencers will be phased out in mid-2016

^b^adopted from http://www.illumina.com/systems/miniseq/specifications.html

^c^Adopted from http://www.illumina.com/systems/miseq/performance_specifications.html

^d^Adopted from http://www.illumina.com/systems/nextseq-sequencer/performance-specifications.html

^e^Adopted from https://tools.lifetechnologies.com/content/sfs/brochures/PGM-Specification-Sheet.pdf

^f^Adopted from https://tools.lifetechnologies.com/content/sfs/brochures/CO06326_Proton_Spec_Sheet_FHR.pdf

extracted from museum specimens [87]. The 454 technology is, however, no longer mainstream and Roche announced that 454 sequencers will be phased out in mid-2016.

Recently, benchtop sequencers have emerged that, owing to their compact format, lower set-up and running costs, and faster data turnaround times, have made NGS accessible for routine testing laboratories. The 454 GS Junior System (Roche), the MiSeq and MiniSeq (Illumina®), the NextSeq 500 (Illumina®), the Ion Proton™ System (Ion Torrrent™) and Ion PGM™ System (Ion Torrrent™) have sequencing capacities large enough for most metabarcoding projects (Box 3). Tillmar et al. [23] used to Roche 454 GS Junior system for the detection of animal species using the 16S rRNA gene. The same benchtop sequencer and the 454 GS-Titanium sequencer were used to identify plant and animal species in TMs [21, 22, 119]. Bertolini et al. [120] used the Ion Torrent PGM™ System for the identification of DNA from meat species using 12S and 16S rRNA genes.

The choice of NGS technology for DNA metabarcoding may depend on several parameters such as the barcode length, the number of barcodes used and the number of samples that need to be analysed.

An advantage of Illumina® sequencing is that sequencing data with very low error rates (greater than 0.1 %) are produced, compared to 454 and Ion Torrent™ sequencing [121]. The most common error types on the 454 and Ion Torrent™ platforms are insertions and deletions (indels), in particular when reading homopolymer regions. This results in an overall error rate of approximately 1.5 % [121, 122]. Sequencing errors can lead to spurious identification of species. Bertolini et al. [120] reported that when Ion Torrent data are quality filtered during downstream bioinformatics processing, the error rates do not introduce any bias that could prevent the correct assignment of meat species.

The high output combined with relatively short length have limited the use of Illumina sequencing technology mainly to profiling of bacterial communities using short 16S rDNA hypervariable regions [123, 124]. However, recent developments allowed the MiSeq platform to double the amount of output per flow cell by producing read lengths of 300 bp (Box 3). Because the Illumina platform can generate amplicon sequences in a paired-end format, paired reads can be directly matched and assembled into amplicons of up to ca. 550 bp.

This development has allowed the MiSeq sequencer to compete with 454 sequencing technology as it allows for generating sequence data from barcode regions with sufficient taxonomic resolution for animal and plant species identification.

An important step in the NGS workflow is to generate a library of the amplicons of interest. Fundamental for library construction is the modification of the DNA amplicons into a form that is compatible with the NGS platform to be used. The library is constructed by enzymatically ligating adapter sequences to the DNA amplicons or by adding them by PCR. The adaptors include specific sequences that are required for clonal amplification of the library on a solid surface (bead or glass slide). The choice of these adapter sequences is dictated by the NGS platform (Box 3). The adapter sequences may additionally contain a 6- to 10-nucleotide-long multiplex identifier (MID) that is used to pool amplicons from several independent samples in one run. MIDs are typically added to make more efficient use of the sequencing capacity of the NGS sequencers i.e. the number of reads generated by each NGS technology is usually higher than required per sample. Adapter sequences with different MIDs need to be used for each sample when multiple samples are sequenced in a single NGS experiment. The number of samples that can be pooled depends on (1) the number of available MIDs, (2) the sequencing capacity of the NGS platform, (3) the number of amplicons per sample and (4) the required sequencing depth [125, 126]. After NGS, the resulting combined sequence data from different samples are subsequently sorted in silico by MID using bioinformatics tools.

### Bioinformatics tools

Bioinformatics has played a crucial role in the advancement of metabarcoding. In recent years, many bioinformatics tools have been developed and are constantly being improved to efficiently and effectively perform various steps involved in the metabarcoding process. After obtaining NGS data, quality filtering is the first essential step, because it removes erroneous data that may otherwise potentially lead to misidentification of species. Sequencing errors introduced during NGS can be recognized because raw reads have predicted error probabilities for each base indicated by Phred quality scores. Sequence errors can be removed during quality filtering and trimming e.g. by truncating reads at the position where their quality begins to drop. A Phred score of 20, which corresponds to a 1 % error rate in base calling, is often used as a minimum threshold in quality filtering. Bokulich et al. [127] published guidelines for quality-filtering strategies to enable efficient extraction of high-quality data from Illumina amplicon sequencing data. In their studies on TMs, Coghlan et al. [21, 119] used the commercially available software Geneious [128]. Other software tools for quality filtering of reads include PRINSEQ [129] and Trimmomatic [130].

Following quality control, the sequences can either be directly matched to a reference library of DNA barcodes or processed further using clustering analysis. Clustering analysis is often performed to improve throughput by removing redundancy in the data such that the input can be used for the more computationally intensive analysis of assigning taxonomy. Clustering methods group reads into operational taxonomic units (OTUs) based on their similarity to other sequences in the samples, and from which representative or consensus sequences are selected. Commonly used clustering algorithms are CD-HIT [131], BlastClust [132] and UPARSE [133]. An OTU is commonly defined as a cluster of reads with 97 % similarity, which would be considered as belonging to a unique species according to the DNA barcoding standard [57]. However, the traditionally used 97 % similarity threshold is only an approximation. Sometimes two closely related species may have identical barcode sequences [134] or conversely single species may have two or more copies of a DNA barcode marker that differ by more than 3 % [135].

Next, tree-based methods and similarity-based methods are most commonly used for assigning query sequences to taxonomy. Tree-based methods assign query sequences to species on the basis of their membership of clusters (or clades) in a barcode tree. This approach is usually based on neighbour joining (NJ) developed by Saitou and Nei [136], and is implemented in BOLD by Ratnasingham and Hebert [41]. The underlying assumption in NJ barcode matching is that distinct species form discrete clusters in an NJ tree [57]. For identification, query sequences are induced in the NJ tree to see which cluster they appear in. Similarity-based BLAST (Basic Local Alignment Search Tool [132]) is probably the most widely used method for classifying DNA sequences in practice. BLAST aligns the query sequence against those present in a selected target database using nearly exact matches of short nucleotide strings (e.g. 10 nucleotides). A similarity score is computed from the portion of the query aligned to the reference sequence. The reference sequence(s) with the highest similarity score is (are) presented along with an indication of the Expect value (E-value), which is the number of hits one can “expect” to see by chance when searching a database of a particular size.

A number of dedicated software pipelines exist that allow processing of metabarcoding data sets followed by taxonomic annotation, including jMOTU and Taxonerator [137], CLOTU [138], QIIME [139], Mothur [140] and UPARSE [133]. These software tools have been developed for studying microbial communities using the 16S rRNA gene fragment, but they can also be used for metabarcoding samples containing plants and animals [21, 141]. The HTS barcode checker pipeline is an application for automated processing of NGS data to determine whether these contain DNA barcodes obtained from species listed on the CITES appendices [16]. DNA metabarcodes are automatically converted into taxonomic identifications by matching with names on the CITES appendices. By inclusion of a blacklist and additional names database, the HTS barcode checker pipeline prevents false positives and resolves taxonomic heterogeneity.

In DNA metabarcoding, the availability of curated reference databases is of major importance to the assignment of sequences to species. A prerequisite is that reference database should contain accurate sequences that are correctly assigned to taxa with adequate sampling and taxon coverage to fully evaluate both the intraspecific and interspecific variations. Unbalanced representation of certain species, which is expected when dealing with CITES species, may greatly affect the analysis. Currently, there are many barcoding campaigns initiated by iBOL to generate DNA barcode data from well-identified and vouchered samples (Box 1). Worldwide sequencing efforts have already resulted in more than 2 million COI records from nearly 170,000 species in BOLD. The Barcode Index Number System (BINs) introduced by BOLD is an online framework that automatically clusters animal COI barcode sequences, generating a wiki Web page for each cluster [142]. Since clusters show high concordance with species, the framework can be used to verify species identifications as well as to document potential new animal species without taxonomic information. BOLD has already reached a good level of standardization and accuracy in terms of the identification of animals but the situation for plants is quite different. The debate about the correct marker(s) to be used as universal barcode has led to a delay in the introduction of plant sequences in the BOLD database [26]. There is also valuable sequence data archived by the International Nucleotide Sequence Database Collaboration (INSDC; www.insdc.org), which, besides the COI region, is particularly extensive for nrITS and cyt*b* (Box 2). However, many of the existing INSDC sequences lack validation in the form of voucher information, making it difficult to detect and remove misidentified specimens or contaminated sequences. Currently the use of local curated reference data sets is often preferred when DNA barcoding is used in plants []. Luo et al. [86] developed a custom DNA barcoding database for medicinal plant materials, and it accepts plastid DNA markers and nuclear nrITS regions as input (www.cuhk.edu.hk/icm/mmdbd.htm). Furthermore, an online identification module for herbal plant materials has been developed (www.tcmbarcode.cn), which is based around a selection of nrITS2 and *psb*A-*trn*H barcodes from selected medicinal species and their adulterants, substitutes and closely related species.

Non-exhaustive list of software available for DNA metabarcoding

**Table:** 

	Description	Reference
Software for quality filtering of reads		
PRINSEQ	Application for filtering, reformatting and quality trimming of metagenomic datasets. The software is publicly available through a user-friendly Web interface and as stand-alone version	Schmieder and Edwards [129] http://edwards.sdsu.edu/cgi-bin/prinseq/prinseq.cgi
Geneious	Commercially available suite of molecular tools	Kearse et al. [128] www.geneious.com
Trimmomatic	A flexible read trimming tool for Illumina NGS data	Bolger et al. [130] http://www.usadellab.org/cms/?page=trimmomatic
Software for cluster analysis of reads		
CD-HIT	A fast program for clustering of next-generation sequencing data. The software is publically available through a user-friendly interface and as stand-alone version	Fu et al. [131] http://weizhongli-lab.org/cdhit_suite/cgi-bin/index.cgi
BLASTclust	A program to make non-redundant sequence sets	Altschul et al. [132] http://toolkit.tuebingen.mpg.de/blastclust
Software for assigning reads to taxonomy		
BOLD identification	Species identification system of the Barcode of Life Data Systems (BOLD)	Ratnasingham and Hebert [41] http://www.boldsystems.org/
BLAST	The Basic Local Alignment Search Tool (BLAST) finds regions of local similarity between sequences. The program is publically available through a user-friendly Web interface and as stand-alone version	Altschul et al. [132] http://blast.ncbi.nlm.nih.gov/Blast.cgi
HTS-barcode-checker	A tool for automated detection of illegally traded species from high-throughput sequencing data	Lammers et al. [16] https://github.com/naturalis/HTS-barcode-checker
Software pipelines for DNA metabarcoding		
jMOTU and Taxonerator	Software for turning DNA barcode sequences into annotated OTUs	Jones et al. [137]
QIIME	Quantitative Insights Into Microbial Ecology: bioinformatics pipeline for microbiome analysis from raw DNA sequence data	Caporaso et al. [139] http://qiime.org/
CLOTU	Software for processing amplicon reads followed by taxonomic annotation	Kumar et al. [138]
UPARSE	Pipeline for clustering NGS amplicon reads into OTUs	Edgar et al. [133] http://drive5.com/uparse/
Mothur	Open-source, platform-independent, community-supported software for describing and comparing microbial communities	Schloss et al. [140] http://www.mothur.org/

### Outlook

Next-generation sequencing of DNA barcodes, commonly referred to as DNA metabarcoding, is more and more becoming a standard approach for the simultaneous identification and detection of multiple species in complex samples. The approach is similar for both species identification to prevent food fraud and for tracing possible cases of illegal trade of CITES species. A large variety of informative barcodes and mini-barcodes in both the animal and plant area is available, potentially allowing for a clear-cut identification of species present in a sample of interest. However, comprehensive identification of (endangered and/or protected) species in complex forensic samples is not yet fully feasible at this moment. This is due to a number of reasons. In the first place, no truly universal DNA isolation method is available for all the different matrixes seized by the customs and CITES authorities. In-house-developed protocols or commercially available kits or a combination of both are typically assessed in an attempt to obtain amplifiable DNA from forensic samples, which will increase time and cost. In many cases, the poor success of extraction and PCR amplification of DNA from forensic samples hinders effective identification of species. Accordingly, systematic studies are needed to optimise DNA isolation methods and efficiency to satisfy the stakeholders’ needs, which are to obtain a robust and rapid DNA isolation method that can be applied across a wide range of (wildlife) forensic samples and one that would maximize DNA purity and yield, whilst reducing any further DNA damage.

Secondly, forensic samples are often heavily processed and may contain severely fragmented DNA, thus hampering the ability to PCR amplify full-length barcodes. In such cases, mini-barcodes are often the only alternative, but these do not always provide species-level resolution, and truly universal primers for mini-barcode amplification have been found difficult to design. Universal primers should be used that minimize PCR bias caused by variable primer–template mismatches across species to ensure that all species can be detected [28, 29]. Several mini-barcodes have been proposed, but especially for plants no universal mini-barcode standard to provide species-level resolution has so far been adopted. The power of DNA metabarcoding is that a panel of different barcodes and mini-barcodes can efficiently be analysed in parallel. Such a strategy will provide improved resolution at the species level when some barcodes fail to resolve, while verifying species with multiple barcodes contributes to enhanced quality assurance.

Thirdly, the current underrepresentation of DNA barcodes from species protected under CITES and closely related species critically hampers their identification. This will improve as DNA barcoding campaigns continue, in particular through initiatives such as the Barcode of Wildlife Project (BWP; www.barcodeofwildlife.org). The latter project aims to construct a public DNA barcode reference library for 2000 endangered plant and animal species, thereby paving the way for the use of DNA barcodes in a court of law to provide strong evidence against those involved in poaching and trafficking of species protected by CITES.

Finally, it will be necessary to develop and validate bioinformatics pipelines for the detection and identification of endangered species using DNA metabarcoding strategies. Several dedicated software tools have been developed, but there is a need to validate pipelines for clustering of reads into OTUs, using benchmarked algorithms for quality control, de-noising, chimera removal and OTU picking.

## Conclusion

The DNA metabarcoding approach holds great promise for detecting and identifying endangered plant and animal species in complex forensic samples. However, validation of the approach should be performed before DNA metabarcoding can be applied in a routine setup. By making use of DNA-barcoded reference species in well-characterized complex products or as internal controls in real-life samples, one can assess whether the DNA metabarcoding procedure is able to accurately and concurrently identify various target plant and animal species. Only when DNA metabarcoding has been demonstrated to be robust and transferable across laboratories can the method truly be implemented in routine testing. In that sense, we are just at the beginning of exploring the broad applications of DNA metabarcoding to reveal the composition of complex products in the light of, for instance, food fraud and the illegal trading of endangered plant and animal species.
